# A risk signature based on endoplasmic reticulum stress-associated genes predicts prognosis and immunity in pancreatic cancer

**DOI:** 10.3389/fmolb.2023.1298077

**Published:** 2023-11-29

**Authors:** Haofei Chen, Ning Xu, Jia Xu, Cheng Zhang, Xin Li, Hao Xu, Weixiong Zhu, Jinze Li, Daoming Liang, Wence Zhou

**Affiliations:** ^1^ The Second Clinical Medical School, Lanzhou University, Lanzhou, China; ^2^ Department of General Surgery, Lanzhou University Second Hospital, Lanzhou, China; ^3^ The Second Affiliated Hospital of Kunming Medical University, Kunming, China; ^4^ Wuhan Blood Center, Wuhan, China; ^5^ Department of General Surgery, The First Hospital of Lanzhou University, Lanzhou, China; ^6^ Department of Gastrointestinal Surgery, The Third People’s Hospital of Hubei Province, Wuhan, China

**Keywords:** ER stress, immune microenvironment, prognosis, bioinformatics, pancreatic cancer

## Abstract

**Introduction:** The involvement of endoplasmic reticulum (ER) stress in cancer biology is increasingly recognized, yet its role in pancreatic cancer (PC) remains unclear. This study aims to elucidate the impact of ER stress on prognosis and biological characteristics in PC patients.

**Methods:** A bioinformatic analysis was conducted using RNA-seq data and clinicopathological information from PC patients in the TCGA and ICGC databases. The ER stress-associated gene sets were extracted from MSigDB. ER stress-associated genes closely linked with overall survival (OS) of PC patients were identified via log-rank test and univariate Cox analysis, and further narrowed by LASSO method. A risk signature associated with ER stress was formulated using multivariate Cox regression and assessed through Kaplan-Meier curves, receiver operating characteristic (ROC) analyses, and Harrell’s concordance index. External validation was performed with the ICGC cohort. The single-sample gene-set enrichment analysis (ssGSEA) algorithm appraised the immune cell infiltration landscape.

**Results:** Worse OS in PC patients with high-risk signature score was observed. Multivariate analysis underscored our ER stress-associated signature as a valuable and independent predictor of prognosis. Importantly, these results based on TCGA were further validated in ICGC dataset. In addition, our risk signature was closely associated with homeostasis, protein secretion, and immune regulation in PC patients. In particular, PC microenvironment in the high-risk cluster exhibited a more immunosuppressive status. At last, we established a nomogram model by incorporating the risk signature and clinicopathological parameters, which behaves better in predicting prognosis of PC patients.

**Discussion:** This comprehensive molecular analysis presents a new predictive model for the prognosis of PC patients, highlighting ER stress as a potential therapeutic target. Besides, the findings indicate that ER stress can have effect modulating PC immune responses.

## 1 Introduction

Pancreatic cancer (PC) is a relatively frequent malignant disease in digestive system, which mostly occurs as pancreatic adenocarcinoma. According to data from the GLOBOCAN, there exists an estimated 459,000 newly diagnosed cases all over the world with an upward trend (Globocan, 2020). Adding to the woes, the mortality rate of PC is almost equal to the morbidity rate, putting it the seventh cause in terms of cancer-associated mortality (Cai et al., 2021). PC has been recognized as a silent disease that hardly presents any clinical manifestations at the early stage. Most PC patients are at an advanced or metastatic stage and lost the opportunity of surgical treatment, when firstly diagnosed (Mizrahi et al., 2020). The advent of intraoperative revascularization and chemotherapy have led to some success in PC treatment (Conroy et al., 2018; Neoptolemos et al., 2018; Sohal et al., 2020). However, the sobering reality is that the overall 5-year survival rate of PC patients is still below 10% (Zeng et al., 2018; Siegel et al., 2020). Worse still, the clinical outcome of PC patients differs largely from a few months to several years. Traditional clinical indicators such as the AJCC stage have poor performance in predicting the survival time for PC, and it is hard for doctors to establish effective therapeutic strategies for PC patients. Thus, it is imperative and urgent to develop novel tools of predicting prognosis so as to provide effective and individualized therapies for PC patients.

Endoplasmic reticulum (ER) is the largest organelle characterized by a high dynamic sac or tube membrane structure that emerges from the nucleus (Schwarz and Blower, 2016). ER plays a vital role in proteostasis due to the exquisite activities of ER-assisted folding (Wiseman and Balch, 2005), ER-associated degradation (Allison, 2021), and COPⅡ export pathway (Zanetti et al., 2011). Abnormal accumulation of misfolded or unfolded protein and alteration of Ca^2+^ concentration will provoke ER stress in eukaryotic cells, subsequently triggering unfolded protein reaction (UPR) that is a homeostatic mechanism mainly mediated by three transmembrane proteins deemed as sensors of ER stress: protein kinase RNA-like ER kinase (PERK), inositol-required enzyme 1 (IRE1α) and activating transcription factor 6 (ATF6) (Hetz et al., 2020). A variety of perturbations in the tumour microenvironment (TME), such as hypoxia, deprivation, acidosis, and accumulation of reactive oxygen species (ROS), which is aggravated by oncogenic events in cancer cells, have been confirmed to drive persistent ER stress. In turn, signal transduction through ER sensors can not only induce UPR but also modulate UPR-independent transcriptional and metabolic pathways, resultantly contributing to tumour initiation, progression, immune evasion, and chemoradiotherapy resistance (Chen and Cubillos-Ruiz, 2021). Prior research has elucidated that ER stress exhibits a significant correlation with the survival outcomes in patients with osteosarcoma (Shi et al., 2022), colon cancer (Liu et al., 2018), renal carcinoma (Zhang et al., 2022), glioma (Zhang et al., 2021). In particular, recent studies show that ER stress can promote stemness and chemoresistance of PC by interfering fatty acid metabolism of tumour cells (Tadros et al., 2017; Dauer et al., 2019). Additionally, hypoxia-induced ER stress has been demonstrated to facilitate PC metastasis by inducing release of vascular endothelial growth factor A (Kong et al., 2015).

A body of work has indicated that ER stress can have effect modulating tumoural immune responses. The study by Pommier et al. unveils that ER stress can induce PC cell dormancy and downregulate the expression of major histocompatibility complex (MHC) class I on PC cells to escape from immune killing, consequently leading to tumour distant metastasis (Pommier et al., 2018). In a subsequent investigation on melanoma, it was discovered that the MHC class I polypeptide-related sequence A/B (MICA/B) - the cognate ligands of major NK activating receptor NK group 2 member D (NKG2D), is negatively regulated by ER stress-associated IRE1/XBP1 pathway in human melanoma cell lines (Obiedat et al., 2019). Beyond modulating the expression of immune-related molecules in cancer cells, the tumour cell-intrinsic ER stress can also reprogram the function of immune cells within the surrounding milieu, which facilitates immune evasion. The findings of Song and colleagues discerned that cell-free ascites from ovarian cancer patients instigate ER stress and triggers IRE1α/XBP1 signaling in activated T cells from healthy women; tumour-bearing mice model further showed that the IRE1α/XBP1 signaling in T cells impairs its mitochondrial respiration by regulating glutamine transporter, ultimately attenuating their anti-neoplastic efficacy (Song et al., 2018). This suggested that ER-stressed cancer cells can release additional factors to remodel the tumour milieu. Another compelling evidence (Yuan et al., 2022) showed that ER-stressed human head and neck squamous cell carcinoma cell line HN4 evades immune surveillance by releasing exosome PD-L1 and upregulating the expression of PD-L1 in macrophages to skew macrophages towards a pro-tumoural phenotype. Cubillos-Ruiz et al. revealed that persistent activation of XBP1 in cancer-associated dendritic cells hindered its capacity to instigate anti-tumour T cells, subsequently catalyzing the progression of ovarian malignancies. (Cubillos-Ruiz et al., 2015). Of note, severe ER stress tip the tumour cells towards releasing damage-associated molecular patterns (DAMPs), triggering immunogenic cell death (ICD) (Rufo et al., 2017). A most recent study revealed that B16 melanoma cells release externalized calreticulin (ExoCRT) during severe ER stress, which is subsequently recognized by the NKp46 receptor on NK cell membrane. And the binding of NKp46–CRT curtails B16 melanoma growth and lung metastasis through amplifying NK killing (Sen Santara et al., 2023). Such revelations underscore that ER stress wields multifaceted impacts on tumours, contingent on its intensity and duration in a context-dependent manner. To holistically understand the intricacies of ER stress in PC, we endeavored to devise a prognostic prediction model for PC patients, underpinned by ER stress-related genes, through an exhaustive exploration of large-scale genomic databases.

In current work, we first identified 46 ER stress-associated genes closely linked with OS of 164 PC patients from TCGA by log-rank test and univariate Cox regression, and further narrowed 12 optimal prognostic genes (SERP1, BCL2L1, RNF139, TSPYL2, ERO1A, BFAR, MAP3K5, ARFGAP1, ATP6V0D1, NCCRP1, PLA2G4B, USP 19) to build a risk signature via LASSO method. Next, these 164 PC patients were classified as two groups based on the risk signature score, and better OS in the individuals with low-risk score was observed by the Kaplan-Meier (K-M) survival analysis method. Meanwhile, the receiver operating characteristic (ROC) curves and multivariate hazard regression demonstrated that our ER stress-associated risk signature was a robust and independent prognostic biomarker. Furthermore, we established a nomogram model by integrating our risk signature and several clinicopathological parameters, which shows promise in predicting prognosis of PC patients. In addition, the risk signature based on ER stress-associated genes was tightly correlated with homeostasis, protein secretion, and immune regulation in PC. Particularly, PC microenvironment in high-risk group exhibited a more immunosuppressive status. In summary, this study suggested that ER stress is of much prognostic significance and may serve as promising therapeutic targets in PC.

## 2 Materials and methods

### 2.1 Datasets and samples

The RNA sequencing metrics of PC samples and normal pancreatic tissues and key clinicopathological characteristics were acquired from TCGA, which is considered as a training cohort. Similarly, we procured the transcriptome profile and clinical data from PACA-CA of the ICGC data portal as an external test cohort. All patients enrolled in this study met the following criteria: i availability of matched gene expression data; ⅱ complete clinical information; ⅲ survival time greater than 30 days. We opted to exclude individuals who, post neoadjuvant therapy, succumbed to extraneous factors such as non-cancerous ailments, other malignancies, or surgical complications. Donors’ transcriptome matrix from Genotype-Tissue Expression Project (GTEx) was used for comparing the expression landscape between PC and normal samples. As for the transcriptome profile, probe IDs were mapped to ENSEMBL IDs and gene symbols using the *Annoprobe* package (Xiang and Zeng, 2019) in R, and genes were filtered based on their expression in more than half of the samples. The counts were just used in the differentiated analysis and were log2-transformed in other cases. The genes involved in ER stress were extracted from MSigDB v7.4 in the two GO pathways (Huang et al., 2021). A list containing Pan-cancer Immune Metagenes of 28 immune cells was obtained from a widely acknowledged research (Charoentong et al., 2017) to reveal the PC immune microenvironment landscape.

### 2.2 Identification candidate genes with prognostic potential

For determining the ER stress-related genes that own prognostic significance, the univariate Cox regression and log-rank tests were carried out (Terry and Patricia, 2001; Therneau, 2021) based on the TCGA-PAAD dataset. A *p*-value threshold of 0.05 was instituted as the criterion for statistical significance. Then the candidate genes associated with PC patients’ survival were then selected based on the *p*-values of the above two test results and included in the following analyses.

### 2.3 Establishment and confirmation of the ER stress-associated risk signature

To assess the link of ER stress with OS of PC patients, we tried to build a risk signature according to the ER stress-associated genes. LASSO penalty algorithm avoiding the overfitting concern (Friedman et al., 2010) was employed to further filter prognostic genes associated with ER stress. In the LASSO regression, the optimal tuning parameter was identified by harnessing the minimal lamda value and the random seed is set to 15091102. Subsequently, multivariate Cox analysis was conducted to yield the ER stress-associated risk signature as previously reported (Song et al., 2022). Then, we scored each PC patient based on the risk signature and calculated the optimal cutoff point of the risk scores. PC patients were grouped low- and high-risk clusters. The OS time of the two clusters was compared by K-M analysis. In addition, the ROC curve was adopted for assessing the prognostic accuracy of our risk signature.

### 2.4 Development and assessment of a predictive nomogram

To better predict the prognosis of PC patients, we established a nomogram by integrating the ER stress-associated signature and several key clinicopathological parameters which have been reported elsewhere as risk factors in PC (Mizrahi et al., 2020; Cai et al., 2021).

### 2.5 Functional enrichment analyses

Differentially expressed genes (DEGs) were initially delineated utilizing the DESeq2 algorithm as (Love et al., 2014), with a criterion of |LogFC| ≥1 coupled with an adjusted *p*-value of < 0.05 to signify statistical relevance. Following this, these identified DEGs underwent comprehensive KEGG enrichment and GO analytic evaluations, applying an adjusted *p*-value benchmark of < 0.05 to ascertain statistical noteworthiness. Furthermore, the paramount enriched KEGG pathways and GO terms were delineated using the GOplot visualization tool.

### 2.6 Evaluation of immune cell infiltration landscape

Utilizing the ssGSEA algorithm, we quantified the abundance of TME immune cells based on gene sets delineated by Charoentong et al. (2017). In detail, 782 genes were regarded as the markers for 28 kinds of immune cells to detect the abundance of each kind of immune cell in PC samples. Additionally, the stromal and immune scores in the two different clusters were calculated via the estimate function (Kosuke et al., 2016), estimating tumour purity. Moreover, using the *complot* (Wei and Simko, 2021), we analyzed the correlation between the abundance of these 28 kinds of immune cells and risk scores, with correlation coefficients derived via the Pearson technique.

### 2.7 Relationship between the risk signature and genomic alterations

In consideration of the vital role of tumour somatic mutations in response to the TME and therapy-induced associated stresses that occur during evolution, we also investigate the association of risk signature with the mutation landscape. Somatic mutation data of PC patients in mutation annotation format, which has been processed under mutect software, was acquired at Genomic Data Commons (GDC) Data Portal. A genomic analysis of PC patients was conducted using the maftools. Tumour mutational burden was further estimated using tmb function to analyze the TMB in different groups.

### 2.8 Prediction of chemotherapy sensitivity for PC patients

To further elucidate the nexus between the ER stress-associated risk signature and therapeutic efficacy, we harnessed the Drug Sensitivity in Cancer (GDSC) database, a comprehensive resource boasting transcriptomic profiles of 805 cell lines and accompanying IC50 values for a staggering 198 bioactive compounds. Using *oncoPredict* R package to align PC transcriptome data in TCGA, we calculated the predicted IC50 value for each PC patient for several chemotherapeutic drugs commonly used in PC treatment. Subsequent comparative analysis between high and low-risk cohorts was facilitated using the Wilcoxon test.

### 2.9 Statistical analysis

The continuous data in two clusters were compared by conducting Wilcoxon test. As mentioned above, combined application of log-rank test and univariate Cox analysis were to screen genes linked with the PC patients’ survival. The LASSO penalty algorithm further narrowed prognosis genes associated with ER stress. Independent prognostic factors were further assessed by multivariate analysis, supplemented with Pearson’s correlation to elucidate the linear relationship between risk scores and immune cell abundance.

## 3 Results

### 3.1 Identification of ER stress-associated genes

**FIGURE 1:**
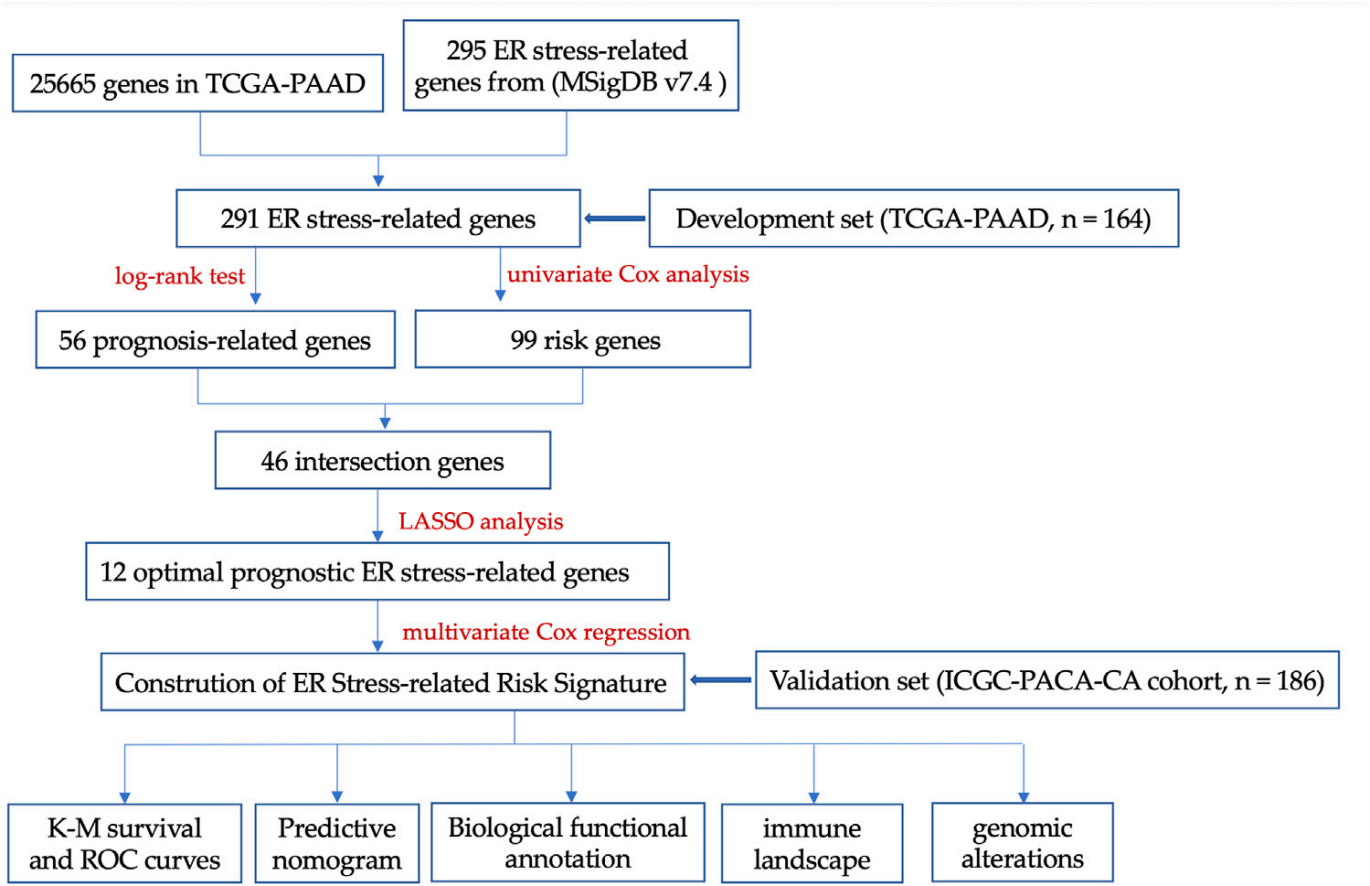
The key design and procedures of this research.

**FIGURE 2:**
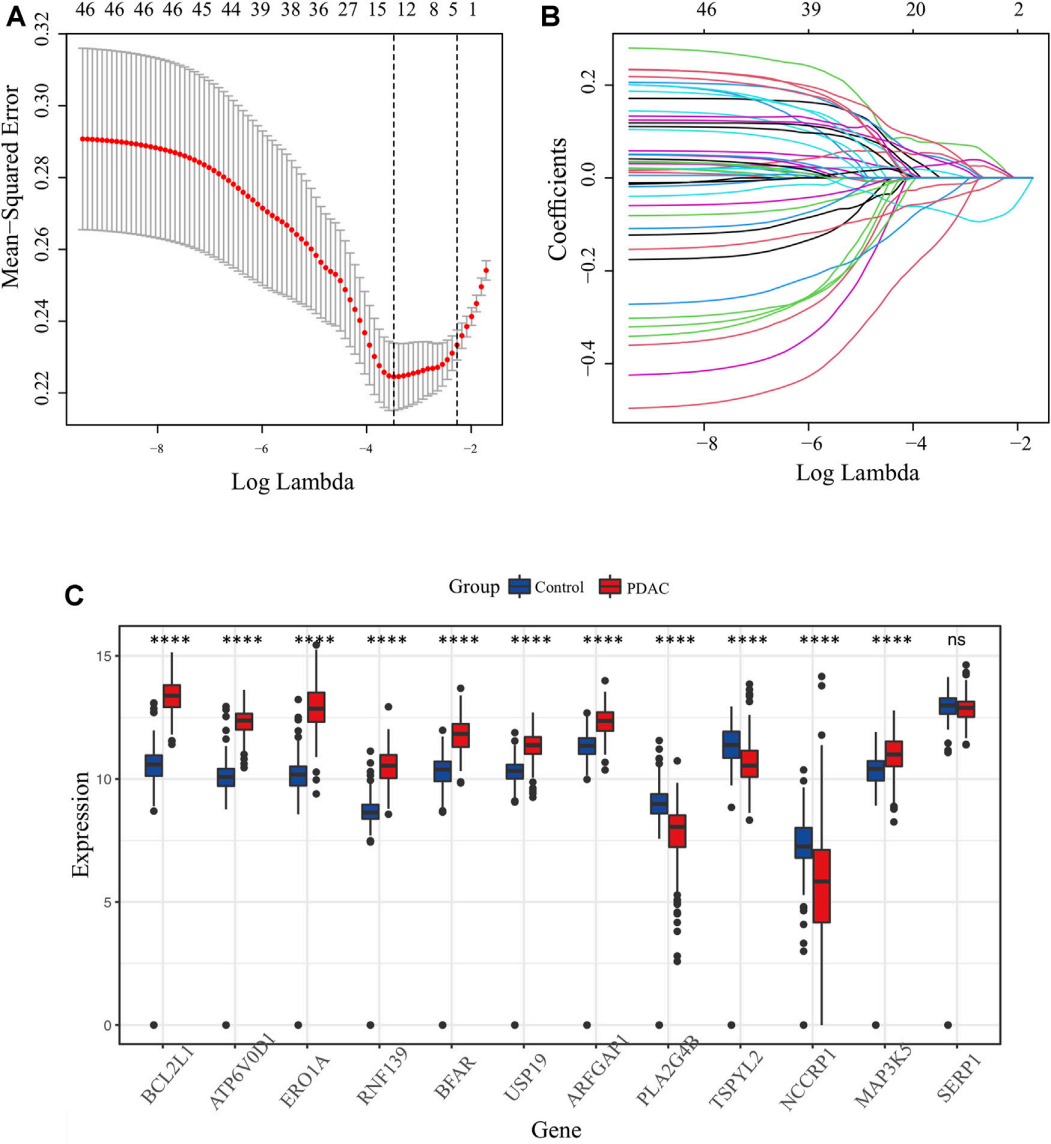
Identification of candidate ER stress-associated genes with prognostic potential. **(A)** Selection of lambda parameters for the LASSO model in TCGA-PAAD dataset. **(B)** Lambda-dependent coefficients for the 46 intersecting ER stress-associated genes. **(C)** Comparative expression profiling of the 12 prime ER stress-associated genes between pancreatic cancer and normal pancreatic tissues.

The key design and procedures of this research were depicted in Figure 1. We finally included 164 PC patients and acquired 295 unique ER stress-associated genes (Supplementary Table S1). Univariate Cox analysis identified 99 risk genes affecting survival (Supplementary Table S2), while log-rank test determined 56 prognostic genes (Supplementary Table S3) within these 295 genes. Furthermore, these 46 intersection genes were considered to have significant relevance to the OS of PC patients and were introduced to LASSO analysis to screen ultimate genes with the best predictive performance. Finally, 12 optimal prognostic ER stress-associated genes with non-zero regression coefficients were determined to construct the risk signature (Figures 2A, B), namely stress-associated endoplasmic reticulum protein 1 (SERP1), B-cell lymphoma 2 like 1 (BCL2L1), ring finger protein 139 (RNF139), testis-specific protein Y-like 2 (TSPYL2), endoplasmic reticulum oxidoreductase 1-α (ERO1A), bifunctional apoptosis regulator (BFAR), Mitogen-Activated Protein 3 Kinase 5 (MAP3K5), ADP-ribosylation factor GTPase activating protein 1 (ARFGAP1), vacuolar proton ATPase subunit d 1 (ATP6V0D1), non-specific cytotoxic cell receptor protein 1 (NCCRP1), phospholipase A2, group IVB (PLA2G4B), ubiquitin specific peptidase 19 (USP 19). The K-M survival plot of every gene for PC patients was shown as Supplementary Figure S1, where the group is based on the best cut-off point for each gene. The mRNA profiles of these 12 genes were also compared between PC tissues and normal pancreatic tissues, and we found that eight of them (BCL2L1, ATP6V0D1, ERO1A, RNF139, BFAR, USP19, ARFGAP1, MAP3K5) were significantly upregulated, three (PLA2G4B, TSPYL2, NCCRP1) were downregulated, and there was no difference in SERP1 expression (Figure 2C).

### 3.2 ER stress-associated risk signature was established, and the low-risk score PC patients owned longer OS time

**FIGURE 3:**
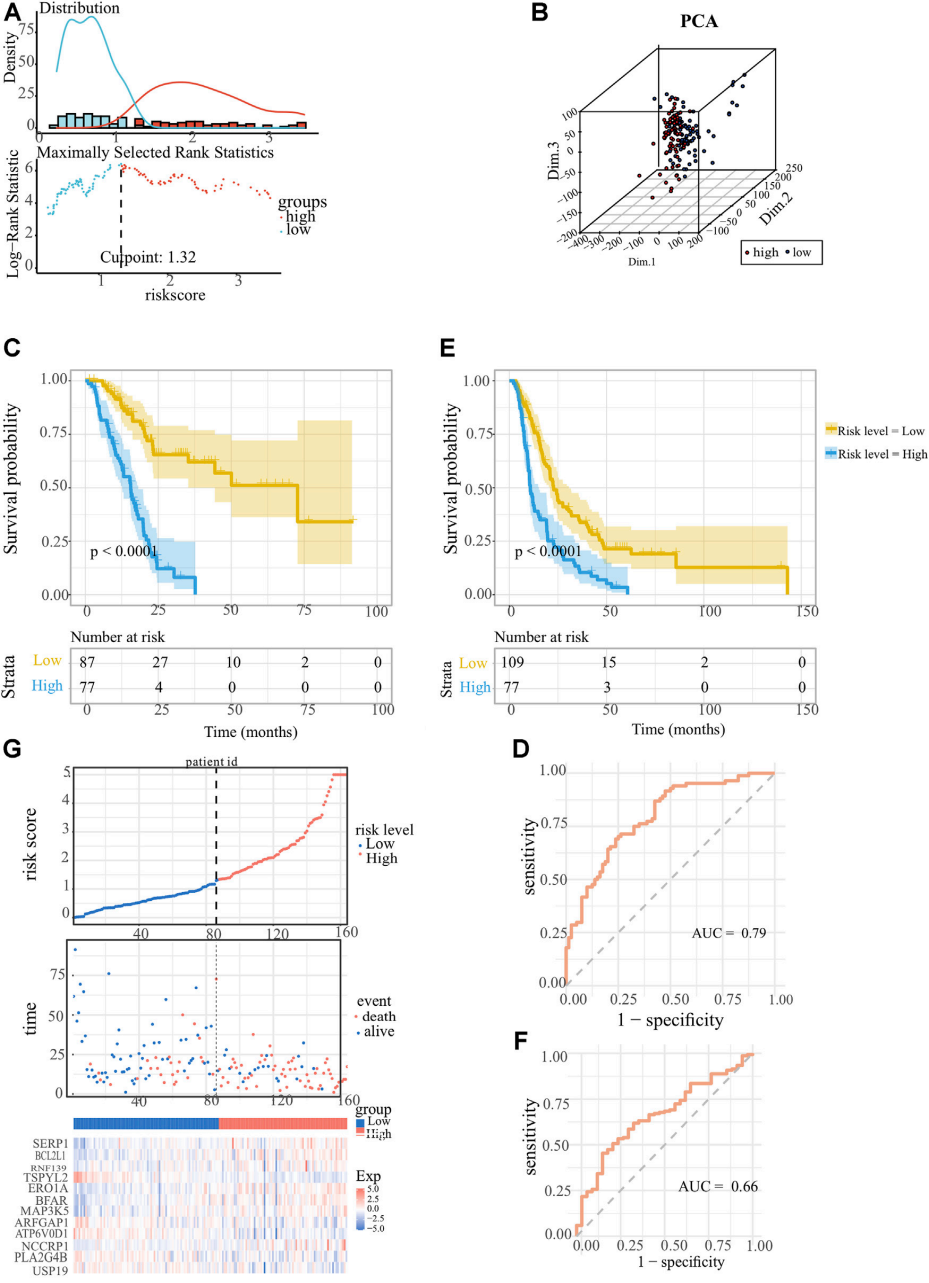
Establishment and confirmation of the ER stress-associated risk signature for pancreatic cancer. **(A)** Optimal cutoff ascertained via survival R package. **(B)** Genomic-wide expression-based Principal Component Analysis (PCA). **(C, E)** Kaplan-Meier survival curves showed indicating adverse overall survival (OS) in PC patients with lower risk indices, as observed in TCGA-PAAD and ICGC-PACA-CA datasets. **(D, F)** Receiver operating characteristic (ROC) curves Receiver Operating Characteristic (ROC) analyses confirming the robustness of the predictive model for pancreatic carcinoma patient survival within the TCGA-PAAD and ICGC-PACA-CA datasets. **(G)** Comprehensive display encompassing risk score distribution, survival status scatter plots, and a heatmap representation of the 12 optimal ER stress-correlated genes.

The ER stress-associated risk signature was established using the above 12 markers, and the risk score equation was: riskscore = 0.813468268 × [(0.496637586 × *SERP1*) + (0.589598808 × *BCL2L1*) + (0.406009527 × *RNF139*) + (−0.521549002 × *TSPYL2*) + (−0.027972152 × *ERO1A*) + (0.323475966 × *BFAR*) + (0.476688839 × *MAP3K5*) + (0.025209694 × *ARFGAP1*) + (-0.92171365 × *ATP6V0D1*) + (0.152614999 × *NCCRP1*) + (0.005952034 × *LA2G4B*) + (-1.026914644 × *USP19*)]. We scored each PC patient with the above signature, and classified 164 PC patients into low-risk group (n = 87) and high-risk group (n = 77) according to the optimal cutoff point (1.322269) of the risk scores (Figure 3A). As Figure 3B showed, the profiles of gene expression in low- and high-risk clusters represented discrepant distribution patterns according to the principal components analysis (PCA), which implied that our ER stress-associated signature was correlated with a whole genome-wide difference. The K-M survival analysis is depicted in Figure 3C. It’s obvious that low-risk score PC patients owned longer OS time (*p* < 0.0001) than those patients with high-risk scores. The area under the ROC curve was 0.79, confirming the prognostic accuracy of our risk signature (Figure 3D). We did the same in the ICGC-PACA-CA cohort to validate the prognostic significance of our ER stress-associated risk signature. Survival analysis also confirmed a statistically significant survival advantage for low-risk score PC patients despite a slight decrease in the AUC value (Figures 3E, F). Moreover, a linked graph of a smooth curve, scatterplot, and heatmap intuitively indicated that as the risk score increases, the survival status of patients with PC from TCGA is more likely to be death and shorter survival time (Figure 3G). Furthermore, the differential expression landscape of these 12 risk factors between the two subgroups was also observed (Supplementary Figure S2).

### 3.3 Establishment of nomogram model integrating ER stress-related risk signature and clinical parameters for predicting prognosis of PC patients

**FIGURE 4:**
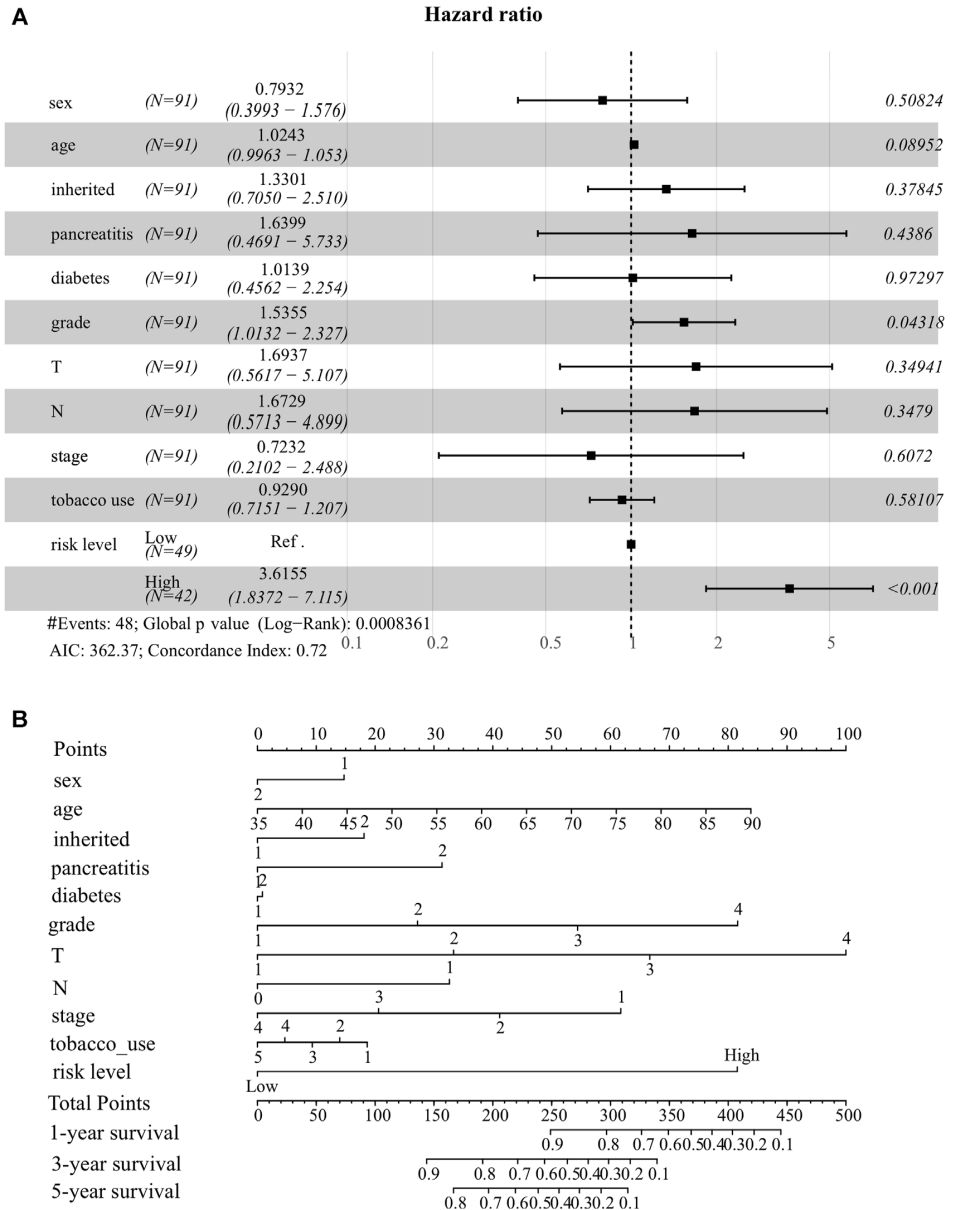
Establishment of nomogram model integrating ER stress-associated risk signature with clinicopathological parameters for prognostic predictions for pancreatic cancer. **(A)** Forest plot showing risk level as an independent prognostic biomarker for pancreatic cancer by multivariate analysis. **(B)** A prognostic nomogram amalgamating the ER stress-associated risk signature and multiple clinical determinants, predicting 1-, 2-, and 3-year overall survival likelihoods in pancreatic cancer patients.

To better predict the survival probability of PC patients, we further built a nomogram based on our ER stress-associated risk signature and several readily available clinical parameters that recognized elsewhere as PC risk factors. Considering the number of patients with complete clinical information in the TCGA-PAAD cohort, our analysis did not include the M stage. Consequently, 91 patients provided complete information contributing to the predicting model. The forest plot showed that risk level and histological grade were independent prognostic indicators (Figure 4A). In particular, the risk level has the most apparent statistical significance (HR = 3.613, *p* < 0.001). Finally, we generated a nomogram model to predict the survival probability for each PC patient with a robust performance (Concordance Index = 0.72), as shown in Figure 4B. Total points can be calculated according to these parameters, and the 1-year, 3-year, and 5-year survival probability can be further obtained based on this total point.

### 3.4 GO and KEGG analyses of the risk signature reveal alteration of the homeostasis and immune signaling pathways

**FIGURE 5:**
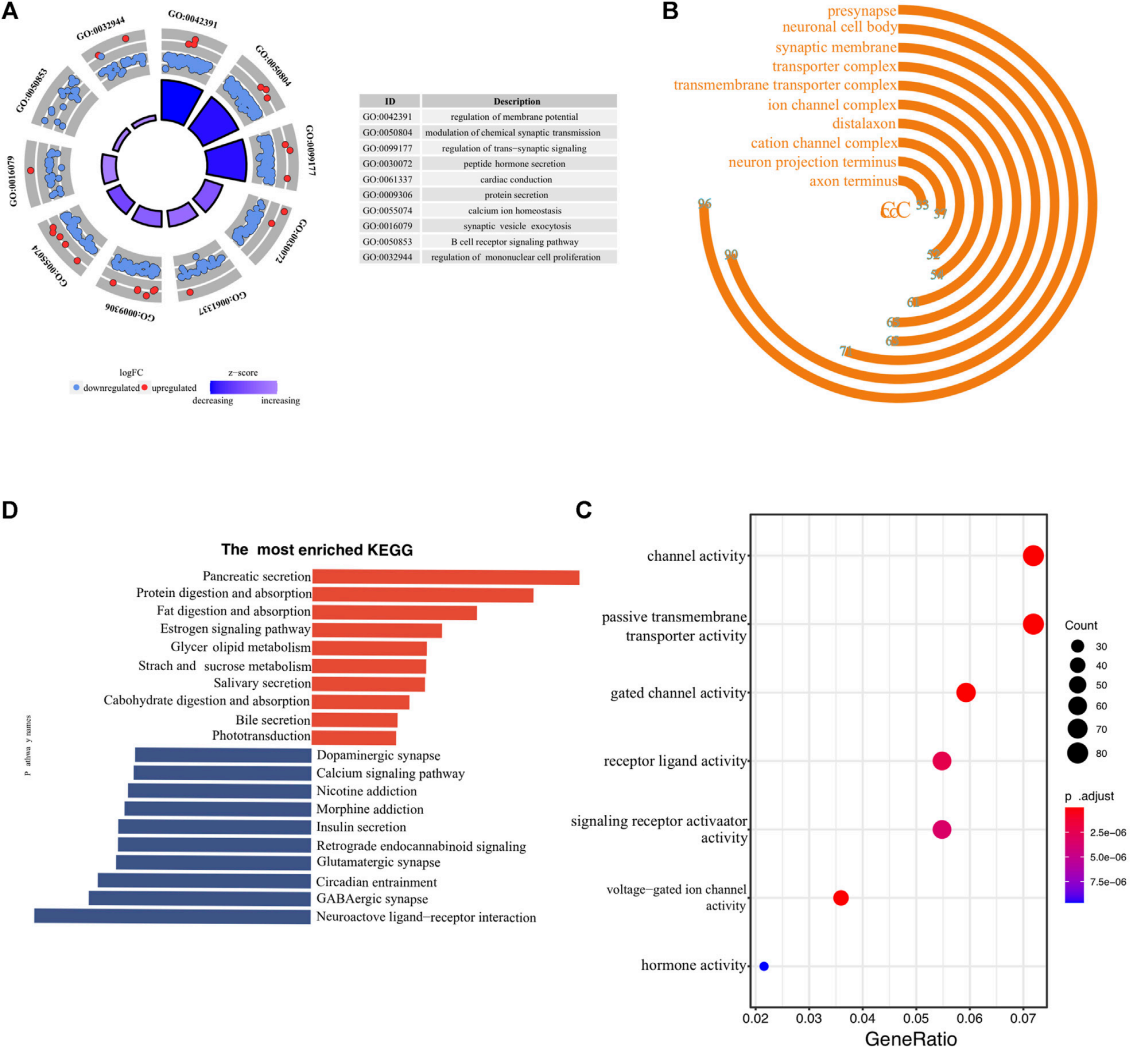
Biological functional annotation of differentially expressed genes between high- and low-risk samples. The front-most enriched biological processes **(A)**, cellular components **(B)**, and Molecular functions **(C)**. **(D)** The most prominently upregulated and downregulated KEGG pathways in the TCGA-PAAD cohort.

To investigate the underlying molecular mechanisms through which ER stress influences the prognosis of pancreatic cancer, we conducted a detailed functional enrichment analysis. Firstly, we identified DEGs between low- and high-risk clusters. A total of 1929 genes were differently expressed, containing 324 high-expressed and 1605 low-expressed genes. The enrichment of biological processes suggested that the ER stress-associated risk signature altered “membrane potential”, “chemical synaptic transmission”, “ trans-synaptic signaling”, “protein secretion” and “B-cell receptor signaling pathway” (Figure 5A). Interestingly, the genes enriched in biological processes terms of the “B cell receptor signaling pathway” were almost downregulated, indicating that high-risk patients may be “colder” regarding the immune microenvironment. The frontmost cellular components were “synaptic membrane,” “presynapse,” “neuronal cell body,” “transport complex,” and “ion channel complex” (Figure 5B). Molecular functions, including “passive transmembrane transporter activity,” “gated channel activity,” and “hormone activity,” were enriched (Figure 5C). More details of GO enrichment analyses can be found in Supplementary Table S4. The top 10 down and upregulated KEGG pathways are shown in bar graphs (Figure 5D). As we can see, the upregulated KEGG pathways mainly involve the exocrine function of the pancreas. Meanwhile, enriched downregulated pathways include “dopaminergic synapse,” “calcium ion signaling,” and “insulin secretion.” Thus, the KEGG and GO analysis results confirmed that the risk signature correlated with homeostasis and emphasized the role of ER status in during PC progression.

### 3.5 Relationship between ER stress-associated risk signature and immune microenvironment

**FIGURE 6:**
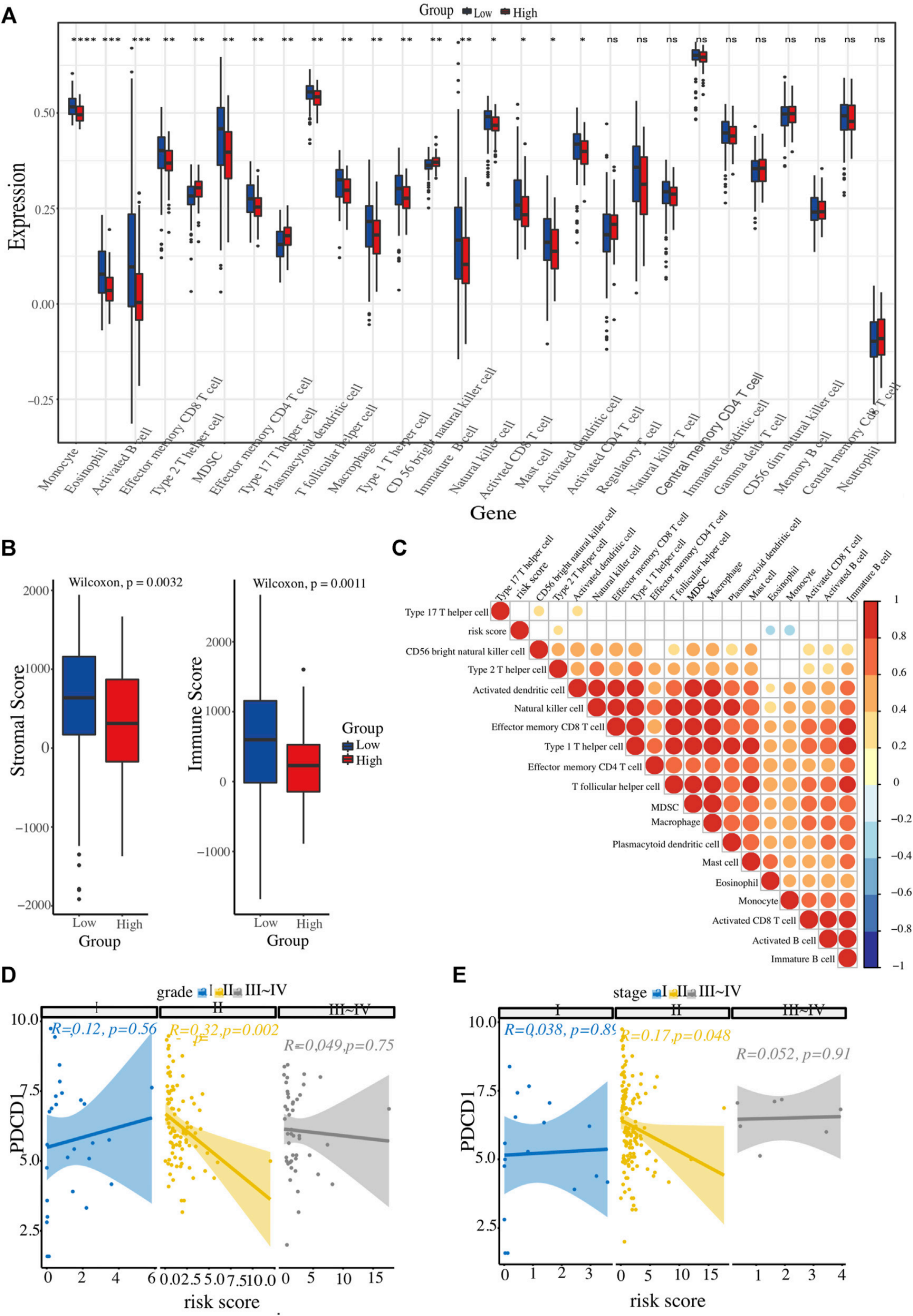
Evaluation of immune cell infiltration landscape. **(A)** Boxplot illustrating differential infiltrations of 28 immune cell subtypes between high- and low-risk groups. **(B)** Comparative evaluation of immune scores and stromal integrity between the two clusters. **(C)** Heatmap representation of risk score associations with 18 immune cell types displaying variable infiltration degrees. **(D, E)** The relationship between ER stress-associated risk score and immune checkpoint (PDCD1), stratified by histopathological grade and AJCC TNM stage, respectively.

The landscape of the immune microenvironment of the two risk clusters were analyzed from the two aspects of immune cell abundance and immune scores. Four kinds of cells, including CD56 bright NK cell, Th2 cell, and Th17 cell, were infiltrated in the high-risk cluster; whereas other 14 types of cells were enriched in l enriched in the low-risk cluster, such as activated CD8^+^ T cells, NK cells, eosinophils, monocytes, activated DCs, as well as macrophages and MDSCs (Figure 6A). In addition, we calculated each PC patient’s stromal and immune scores at different risk levels. Unsurprisingly, the high-risk group got lower scores (Figure 6B). To further explore whether the ER stress-related signature affects PC immune microenvironment, a correlation heatmap was constructed to reflect the correlations between these differentially infiltrated immune cells and risk scores (Figure 6C). All these results demonstrated that the high-risk cluster has a more suppressive immune microenvironment that interacts with the risk signature. Considering the close link between our risk signature and the immune microenvironment and the therapeutic potential of immunotherapy in selected tumours, we also conducted a correlation analysis to identify PC patients who could benefit from immunotherapeutic modalities. Several immunotherapy targets, such as PDCD1, CTLA4, LMTK3, and LAG3, were also expressed in PC tissues. Interestingly, PDCD1 expression level had an inverse correlation with the risk score in PC patients with histopathological grade 2 or AJCC stage Ⅱ (Figures 6D, E).

### 3.6 Association of ER stress-associated risk with genomic alterations

**FIGURE 7:**
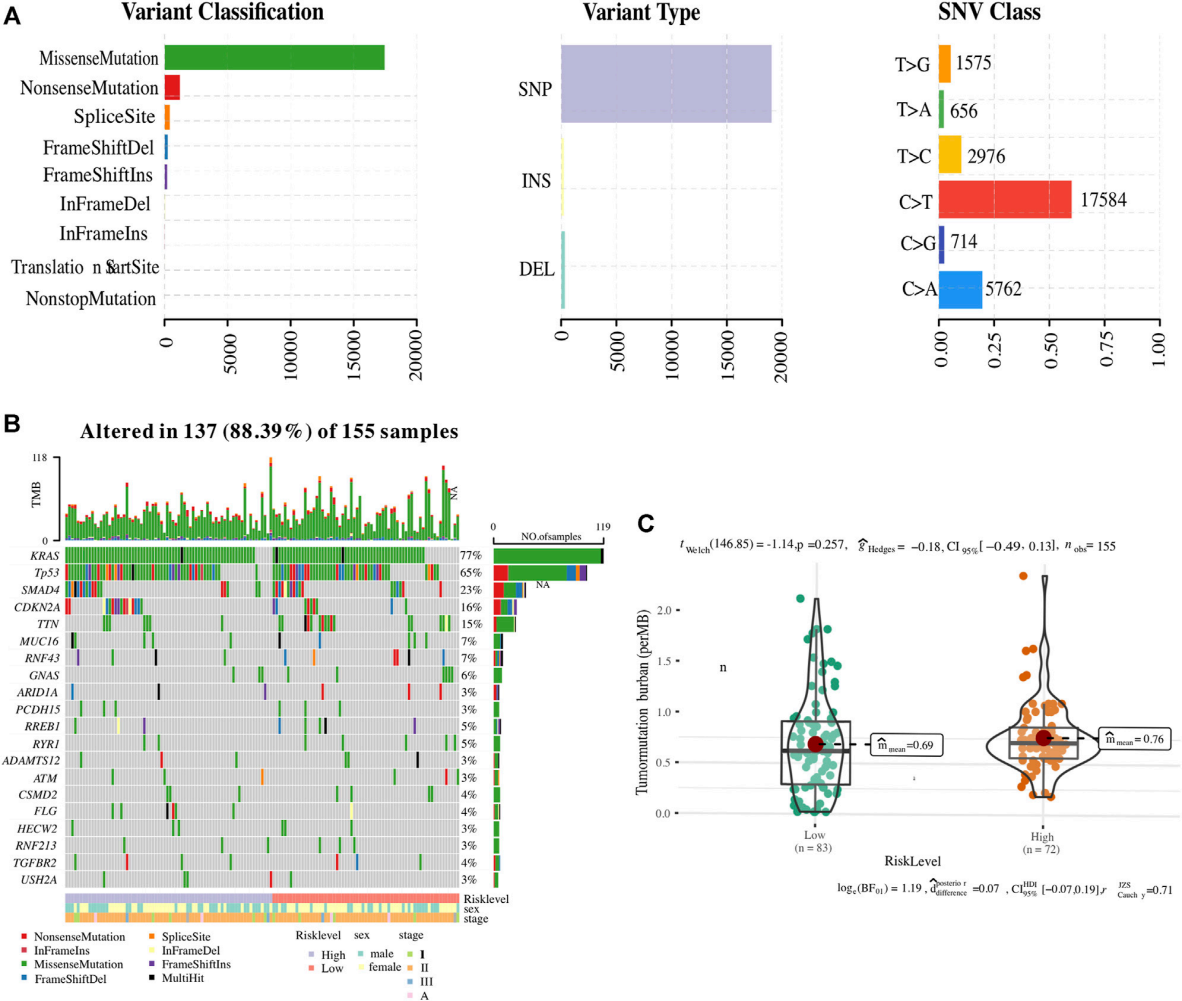
Association of ER stress-related risk signature with genomic alterations. **(A)** The summary of the mutation landscape of pancreatic cancer. **(B)** Waterfall representation highlighting the top 10 mutated genes. **(C)** Comparative tumour mutational burden (TMB) across the two ER stress-associated risk stratifications.

Since somatic mutations are closely related to tumour initiation and progression, an integrated analysis was performed to establish an association of the status of genomic alterations with our ER stress-associated signature in PC. A total of 9,957 genes were mutated among 178 PC samples obtained from the GDC website, of which *KRAS*, *TP53*, *SMAD4*, and *CDKN2A*-the four main drive genes, were the most frequently mutated. The summary of the mutation landscape shows that missense mutation, nonsense mutation, frameshift deletion or insertions, and splice site mutation are the leading fourth variant types (Figure 7A). A waterfall plot depicted the top 10 mutated genes under ER stress signature-based clusters and other clinicopathological groups, and we found that 77% of PC patients experienced *KRAS* mutation (Figure 7B). As for the 12 ER stress-associated genes for constructing the risk model, only *BFAR*, *MAP3K5*, and *ARFGAP1* have missense mutations or frameshift insertions, and *USP19* has multiple mutations (multi-hits). The mutation ratio is less than 1%. In addition, the TMB was also calculated to reveal a possible mechanism for the different immune microenvironments in the two risk groups. However, our result showed no difference in TMB between the two clusters (Figure 7C).

### 3.7 Correlation between ER stress-associated risk signature and therapeutic response

**FIGURE 8:**
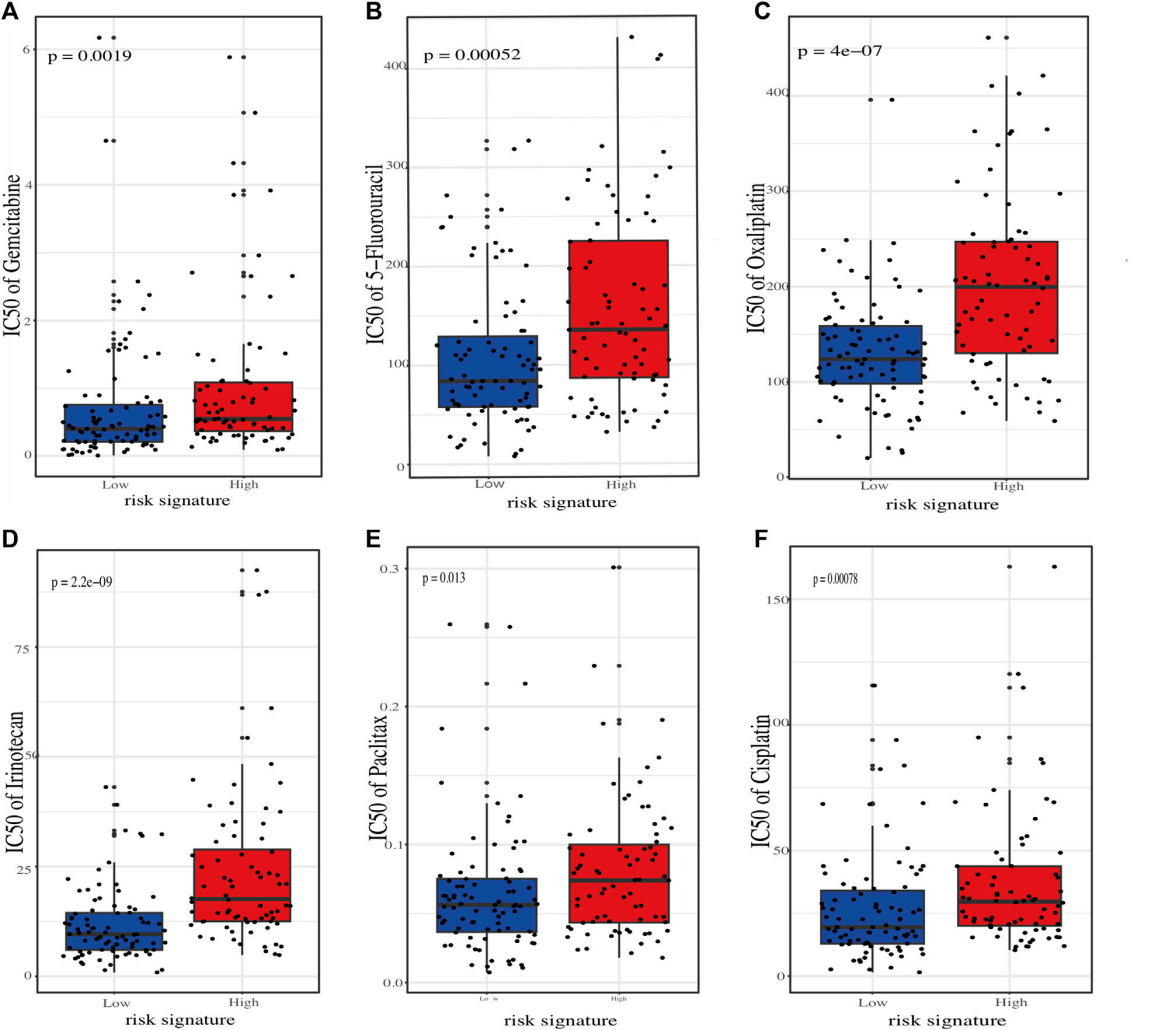
Estimation of chemotherapeutic sensitivity in stratified risk groups. Comparision of the predicted half-maximal inhibitory concentration (IC50) values for six chemotherapeutic agents: gemcitabine **(A)**, 5-fluorouracil **(B)**, oxaliplatin **(C)**, irinotecan **(D)**, paclitaxel **(E)**, and cisplatin **(F)**.

To better connect our ER stress-related risk signature to clinical practice, we employed oncoPredict tool to calculate the IC50 value of several commonly used chemotherapeutic agents for each PC patients. The results showed that the predicted IC50 for agents such as “5-Fluorouracil”, “Oxaliplatin”, “Irinotecan”, “Paclitaxel”, and “Cisplatin” skews higher for the high-risk demographic., indicating that low-risk score patients may be more sensitive to chemotherapy (Figures 8A–F).

## 4 Discussion

The traditional clinical indicators are poor in predicting the survival time of PC, so it is imperative to develop new tools to predict survival and furnish effective individualized treatment for PC patients. Increasing evidence shows that ER stress plays a complex role in PC progression. Hence, we here performed a bioinformatic analysis based on TCGA database to study role of ER stress in prognosis and biological characteristics in PC patients. In this study, we initially established a twelve-gene risk signature in terms of ER stress according to the gene expression counts and corresponding survival time of 164 PC patients from TCGA. The twelve ER stress-related genes enrolled in the risk model could be divided into protective factors (*TSPYL2*, *ARFGAP1*, *ATP6V0D1*, *PLA2G4B*, *USP19*) and pernicious factors (*SERP1*, *BCL2L1*, *RNF139*, *ERO1A*, *BFAR*, *MAP3K5*, *NCCRP1*). The predictive potency of the ER stress-associated risk signature was further validated by another independent PC dataset from ICGC. Survival curve and forest plot demonstrated that our ER stress-associated signature was a valuable and independent prognostic biomarker, according to the ROC curves (AUC was 0.79) and the hazard ratio (HR was 3.613, *p* < 0.001, CI was 0.72). Besides, we constructed a credible nomogram integrated the risk signature and several clinicopathological parameters predicted the OS of individual PC patients. Function annotations elucidate the biological role of ER stress in PC, and the results indicated that the ER stress-related signature has an effect in shaping TME immune cell infiltration.

SERP1 has been uncovered to be overexpressed under stress, which helps to stabilize membrane proteins and functions as a translocon on the ER membrane (Yamaguchi et al., 1999). Evidence shows that SERP1 may act as an oncogene and could be developed as a valuable prognostic biomarker. For instance, a recent research uncovered that SERP1 can facilitate osteosarcoma progression through modulating circ_0085539/miR-526b-5p signalling axis (Liu et al., 2020). Additionally, SERP1 has been reported to maintain the viability of PC cells promote the survival of PC cells by down-regulating apoptosis-related protein SRPRB and activating the NF-κB signalling pathway (Ma et al., 2017). Consistently, our study demonstrated that SERP1 was upregulated in PC and its high expression predicted dismal patient survival prognosis. BCL2L1, also called Bcl-extra (Bcl-x), belongs to the B-cell lymphoma 2 apoptosis family. BCL2L1 translates two antagonistic variants including Bcl-X_S_ and Bcl-X_L_ due to alternative splicing. The short isoform Bcl-X_S_ exhibits pro-apoptosis biological function, whereas the long isoform Bcl-X_L_ contributes to survival of cells. Numerous studies have confirmed that the disequilibrium of BCL2L1 splice is related to the progression of multiple malignancies (Dou et al., 2021). In addition to promoting tumour cell survival, Bcl-X_L_ can also boost metastasis and EMT of pancreatic neuroendocrine tumour cells via epigenetically enhancing TGFβ signalling (Choi et al., 2016). Furthermore, recent evidence shows in melanoma that Bcl-X_L_ can positively regulate the expressions of IL-8 and CCL5, which not only recruit macrophages into tumour sites, but also polarize them into M2 phenotype (Lucianò et al., 2023). In PC, Bcl-XL is overexpressed, and correlated with worse prognosis of patients (Friess et al., 1998). Particularly, Bcl-X_L_ has been identified as a crucial mediator of acquiring radioresistance and gemcitabine resistance (Lee et al., 1999; Thummuri et al., 2022). Similarly, we herein found that BCL2L1 was significantly increased in PC tissues and its high expression predicted unfavourable prognosis of patients. In general, our data and the literature evidence suggest that Bcl-X_L_ may become a valuable prognostic biomarker and serve as a therapeutic target for PC patients.

RNF139, also known as TRC8, encodes a multi-membrane spanning protein resides in ER, and it has been revealed to act as a tumour suppressor. A study reported that RNF139 was significantly downregulated in tongue cancer tissue and experimentally that silencing RNF139 enhanced the viability and aggressiveness of tongue cancer cells (Wang et al., 2017). Intriguingly, our data shows that RNF139 is dramatically upregulated in PC tissues, and low-expressed RNF139 PC patients have shorter survival time, indicating a potential role of RNF139 in propagating PC progression. Hence, the roles of RNF139 in cancer may depend upon the tumour types, and cell-based and animal experiments are warranted to determine the specific roles of RNF139 in PC. ERO1A is an oxidoreductase located in ER lumen. Recently, accumulating data uncovers that ERO1A functions as an oncogene in many types of cancers. According to Yan and colleague, the expression of ERO1A is increased in cholangiocarcinoma and its overexpression predicts poor prognosis of patients (Yan et al., 2019). Zilli et al. (2021) revealed that upregulation of ERO1A significantly promotes primary breast tumour growth and lung metastatic colonization by enhancing HIF1α-VEGFA mediated angiogenesis. Zhang et al. corroborated that overexpression of ERO1A can contribute to tumour progression by accelerating hydrogen peroxide-associated epithelial-mesenchymal transition, and it may be used as a predictor for dismal prognosis in cervical cancer (Zhang et al., 2019). In PC, ERO1A has been demonstrated to boost tumour growth via augmenting aerobic glycolysis of tumour cells (Zhang et al., 2020). In agreement with these evidence, we sighted that mRNA of ERO1A is highly in PC and high ERO1A expression is accompanying worse prognosis. Altogether, ERO1A may be a useful prognostic biomarker and promising therapeutic target for PC patients.

BFAR has been recognized as an inhibitor of apoptosis with an alary helix DNA-binding structure and is tightly linked with tumour progression. Cheng et al. reported that BFAR is upregulated in glioma tissues and glioma patients with lower BFAR expression have worse long-term survival prognosis (Cheng et al., 2020). In gastric cancer, BFAR has been proven to rebound to the tumour metastasis via activating the PI3K/AKT/mTOR signalling axis (Chen et al., 2023). Similar to the previous studies, we observed that BFAR is highly expressed in PC and its increasing expression predicts worse outcomes of PC patients. Thus, BFAR may also act as an oncogene in PC. MAP3K5 has been known as an upstream protein of mitogen-activated protein kinase cascade signalling pathway and plays a vital tumour-suppressive role in many types of cancers. For instance, Cheng et al. showed that overexpression of MAP3K5 promotes PC cell apoptosis via activating the p38/MAPK axis (Cheng et al., 2014). Consistent with this, our data showed that MAP3K5 is upregulated in PC tissues and its high expression predicts poor prognosis of PC patients. NCCRP1 is a type III transmembrane protein containing an antigen recognition motif, also named as FBXO50. Evidence shows that NCCRP1 plays an essential role in tumour development, and whether it acts as a tumour suppressor gene or oncogene depend upon tumour types. A most recent study suggested that NCCRP1 was highly expressed in triple-negative breast cancers (TNBCs) and its overexpression could markedly enhance cell proliferation (Zhou et al., 2022). In gastric cancer, higher NCCRP1 expression is associated with shorter recurrence-free and overall survival time, as it promotes proliferation, movement, and incursion of gastric cancers (Miwa et al., 2017b). Inversely, another study (Miwa et al., 2017a) found that esophageal squamous cell carcinoma patients with lower NCCRP1 expression tended to have an increased risk of disease recurrence and dismal survival prognosis versus those with higher NCCRP1 expression, suggesting a putative tumour-suppressive role of NCCRP1. Notably, when it comes to PC, the roles of NCCRP1 seems rather complex. In our study, we found that NCCRP1 is downregulated in PC tissues compared to normal pancreatic tissues, but the high NCCRP1 expression patients have little survival time. Our paradoxical results may be due to the fact that NCCRP1 in PC tissues is not expressed mainly in tumour cells, but in stroma cells such as immune cells and fibroblasts. More research is thus required to further decipher the biological roles of NCCRP1 in PC and its clinical prognostic value.

USP19 is a kind of deubiquitinating protease. Similar to NCCRP1, USP19 is aberrantly expressed in multiple cancers and may act as a tumour suppressor gene or oncogene, which relies upon the tumour type (Rossi and Rossi, 2022). For example, USP19 was found to be upregulated in TNBC tissues and promote the invasion and metastasis of TNBC (Rossi et al., 2021). Besides, a recent study reported that upregulation of USP19 contributed to tumourigenicity of colorectal cancer cells by stabilizing Survivin protein (Chandrasekaran et al., 2022). On contrast, diminished USP19 expression in ovarian cancer correlates with an adverse prognosis (Kang et al., 2021). Adding to this complexity, (Hu et al., 2020), reported in clear cell renal cell carcinoma that overexpression of USP19 markedly curtails tumour cell proliferation and migration via ERK pathway inactivation. Aligning with these findings, our research discerned that elevated USP19 expression portends a favorable survival outcome in PC, and it is of clinical significance to investigate its specific biological functions and the relevant mechanisms in the progression of PC. TSPYL2 is the nucleosome component of chromatin remodelling (Cardano et al., 2023). Recent work by Pan et al. (2022) recently confirmed that TSPYL2 is downregulated in PC and overexpression of TSPYL2 can effectively overcome gemcitabine resistance in PC cells. Likewise, in this study we also found that TSPYL2 is significantly downregulated in PC and its low expression predicts shorter survival time of PC patients. Within the cohort of ER stress-associated risk signature genes, the roles of PLA2G4B, ARFGAP1, and ATP6V0D1 in human malignancies remain largely unexplored. Our research determined that PLA2G4B exhibits decreased expression in PC tissues, correlating lower expression levels with a more guarded prognosis. In contrast, both ARFGAP1 and ATP6V0D1 manifested heightened expression in PC tissues, resonating with an improved prognostic outlook for PC patients. Future endeavors should be directed towards elucidating the precise biological and clinical implications of PLA2G4B, ARFGAP1, and ATP6V0D1 in the context of PC.

The tumour immune microenvironment is intertwined with PC growth, metastasis, and therapeutic resistance (Bear et al., 2020; Ho et al., 2020; Sherman and Beatty, 2023). Increasing evidence reveals that ER stress can confer immunomodulatory capacity on cancer cells (Cubillos-Ruiz et al., 2017; Di Conza et al., 2023), prompting us to evaluate the correlation of our ER stress-associated signature with the PC immune microenvironment. The functional enrichment analysis showed that ER stress status in PC may have a close relationship to a multiple of immune signalling pathways including T cell proliferation, B cell proliferation and activation, and interleukin-4 production. A body of findings in oncological research indicate that ER stress can have effects on the trafficking of immune cell to the tumour milieu. For instance, Song et al. showed XBP1 is upregulated in T cells isolated from ovarian cancer patient samples in contrast with healthy donor-derived T cells, and the upregulation of XBP1 is correlated with decreased intratumoural T cell infiltration (Song et al., 2018). Work by Harnoss and colleagues demonstrated that in TNBC xenograft, knockout of IRE1α can reduce MDSC infiltration by downregulating the transcription of several genes involved in recruitment, such as PTGS2, CXCL8, and CXCR4 (Harnoss et al., 2020). Histological analyses revealed that in human hepatocellular carcinoma (HCC) specimens, the expression levels of ER stress markers such as BiP, ATF6, PERK, and IRE1α were closely associated with an elevated infiltration of CD68^+^PDL1^+^ macrophages. The elevating PD-L1 expression on macrophages is primarily achieved by exosomes containing miR-23a-3p released from HCC cells subjected to ER stress mediated PTEN/PI3K/AKT pathway, which in turn impacts T cell functions and survival (Liu et al., 2019). In our study, we observed that low-risk cluster has higher accumulation of T cells and NK cells, comparing with high-risk cluster. It has been demonstrated that low infiltration and dysfunction of NK cells in PC tissues are tightly associated with tumour recurrence and survival of patients (Hoshikawa et al., 2018; Xie et al., 2019). As such, our ER stress risk signature could serve as a valuable tool for evaluating NK cell immune status and predicting the efficacy of NK cell-based immunotherapy in PC. Since the cytotoxic and cytokine-producing effector molecules functions of NK cells are governed by a delicate balance between activating and inhibitory receptors, we sought correlations between NK cell ligands and our risk signature, albeit with inconclusive outcomes. Comparative analyses revealed that our high-risk and low-risk cohorts exhibited no discernible disparities in the mRNA expression levels of the MICA/B, cognate ligands of major NK activating receptor NKG2D (Cadoux et al., 2021), and the inhibitory receptors ligands HLA-E (Liu et al., 2023) and CEACAM1 (Park et al., 2020). Interestingly, relative to healthy pancreatic tissue, all the aforementioned molecules exhibited augmented expression in PC. This observation may be attributed to the impairment of IRE1α arm in PC, as it was reported that the MICA/B expression was suppressed by the IRE1α/XBP1 arm of the ER stress response in melanoma cells (Obiedat et al., 2019). Eosinophils, primitive cells of the innate immune system primarily studied in allergic diseases, have recently garnered attention for their role in promoting antitumour immune responses (Grisaru-Tal et al., 2022; O'Flaherty et al., 2017; Blomberg et al., 2023) demonstrated that eosinophils can enhance NK cell chemotaxis and activation via secretion of CCL5, CXCL10 and IL-12. Moreover, colorectal cancer-infiltrated eosinophils have been shown to promote IFN-γ and TNF-α production by CD8^+^ T cells via the GM-CSF-IRF5 signalling axis, thereby augmenting antitumour immune responses (Arnold et al., 2020). Intriguingly, our findings revealed a predominant concentration of tumour-infiltrating eosinophils in the low-risk cluster compared to its high-risk counterpart, potentially elucidating the abundant occurrence of NK and T cells within the low-risk group. It's hypothesized that ER stress might compromise the antitumour efficacy of NK and T cells by obstructing eosinophil recruitment and activation. Both macrophages and MDSCs, pivotal myeloid suppressive entities, can debilitate NK and T cell functionality, facilitating tumour immune evasion (Sui et al., 2022; Cassetta and Pollard, 2023). Our observations indicate an inverse relationship between MDSCs and ER stress scores, presenting an apparent paradox. Given the pronounced presence of NK and T cells within low-risk strata, this data intimates potential regulatory feedback exerted by MDSCs that modulates NK- and T-cell-mediated antitumour immune responses. Thus, ER stress’s influence on the TME seems intricate, making targeted interventions demanding. Therefore, the effect of ER stress on the TME may be multifaceted and targeting ER stress remains challenging.

In summary, we established a 12-gene ER stress-associated risk signature, holding promise as a prognostic tool for PC patients. Importantly, this prognostic risk signature is positively correlated with an immunosuppressive state in PC, indicating its potential of guiding clinical decision making in the immunotherapy of PC. The analyses of functional enrichment and quantification of the immune infiltration landscape yielded essential findings for initiating future research on the mechanisms underlying ER stress-related genes and tumour immunity in PC. Nonetheless, it's crucial to highlight that our investigation relied solely on transcriptomic data from surgical biopsies, so further clinical trials and experimental validations are required to confirm our findings.

## Data Availability

Publicly available datasets were analyzed in this study. This data can be found here: The Cancer Genome Atlas (https://xenabrowser.net/), International Cancer Genome Consortium (https://icgc.org), and Molecular Signature Database v7.4 (https://www.gsea-msigdb.org/). The full original source data used in this study has been downloaded from the above public datasets and uploaded to jianguoyun along with codes. Detailed information can be found here: “https://www.jianguoyun.com/p/DYpnVlIQ4qX6CxjQ3ZoFIAA.”
