# Supplementary material for: A risk signature based on endoplasmic reticulum stress-associated genes predicts prognosis and immunity in pancreatic cancer
Source: Front Mol Biosci. 2023 Nov 29;10:1298077. doi: 10.3389/fmolb.2023.1298077 (PMC10721979; doi:10.3389/fmolb.2023.1298077)
Supplement: Supplementary file 2 [file DataSheet1.docx]

Supplementary Material

A risk signature based on endoplasmic reticulum stress-associated genes predicts prognosis and immunity in pancreatic cancer

Haofei Chen^1,2†^, Ning Xu^3†^, Jia Xu^4†^, Cheng Zhang^3†^, Xin Li^2^, Hao Xu^5^, Weixiong Zhu^1^, Jinze Li^6*^, Daoming Liang^3*^ and Wence Zhou^1,2*^

# Supplementary Figures


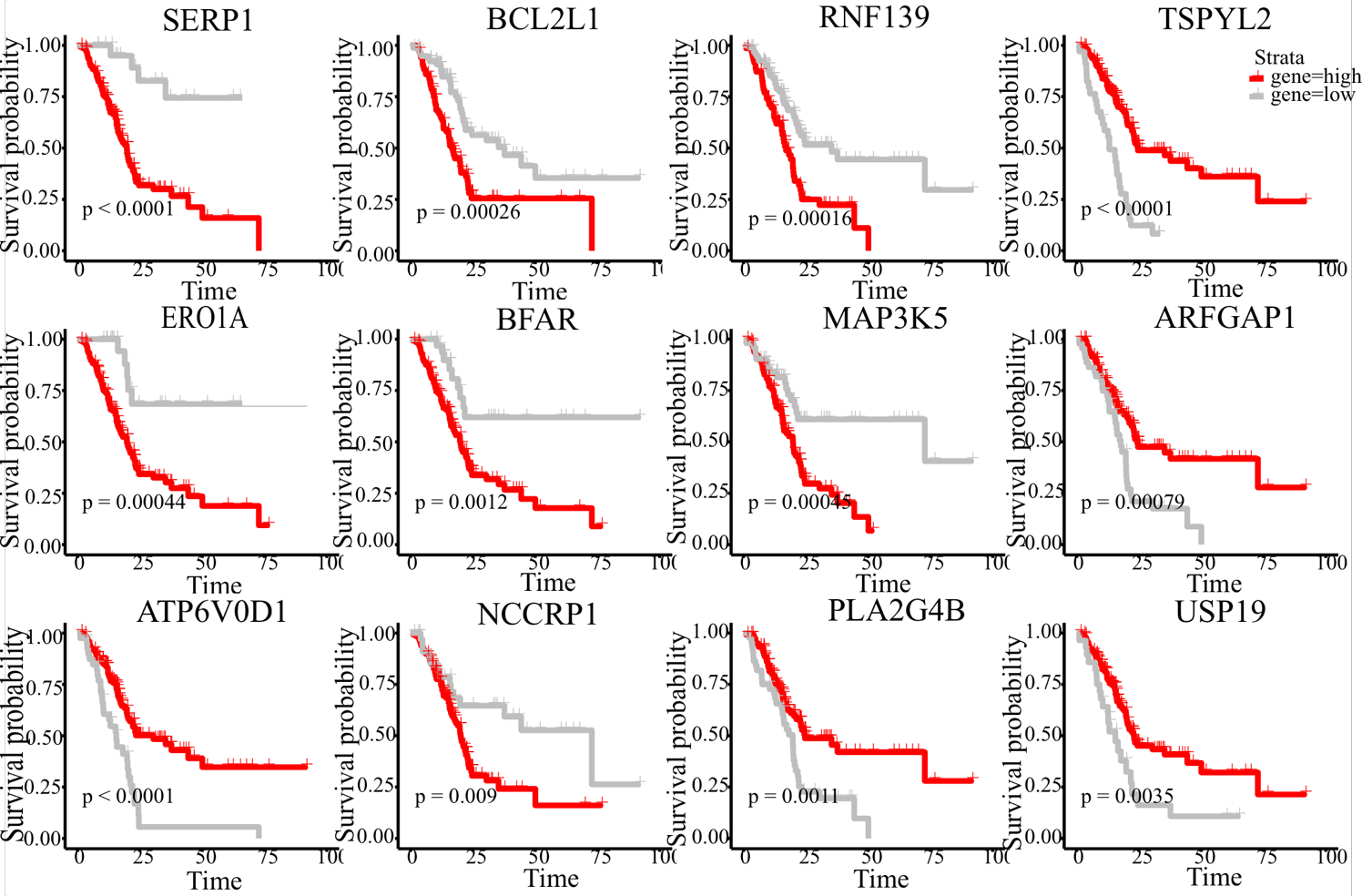


**Supplementary Figure 1.** The prognostic value of our 12 ultimate ER stress-associated genes in the TCGA for pancreatic cancer.


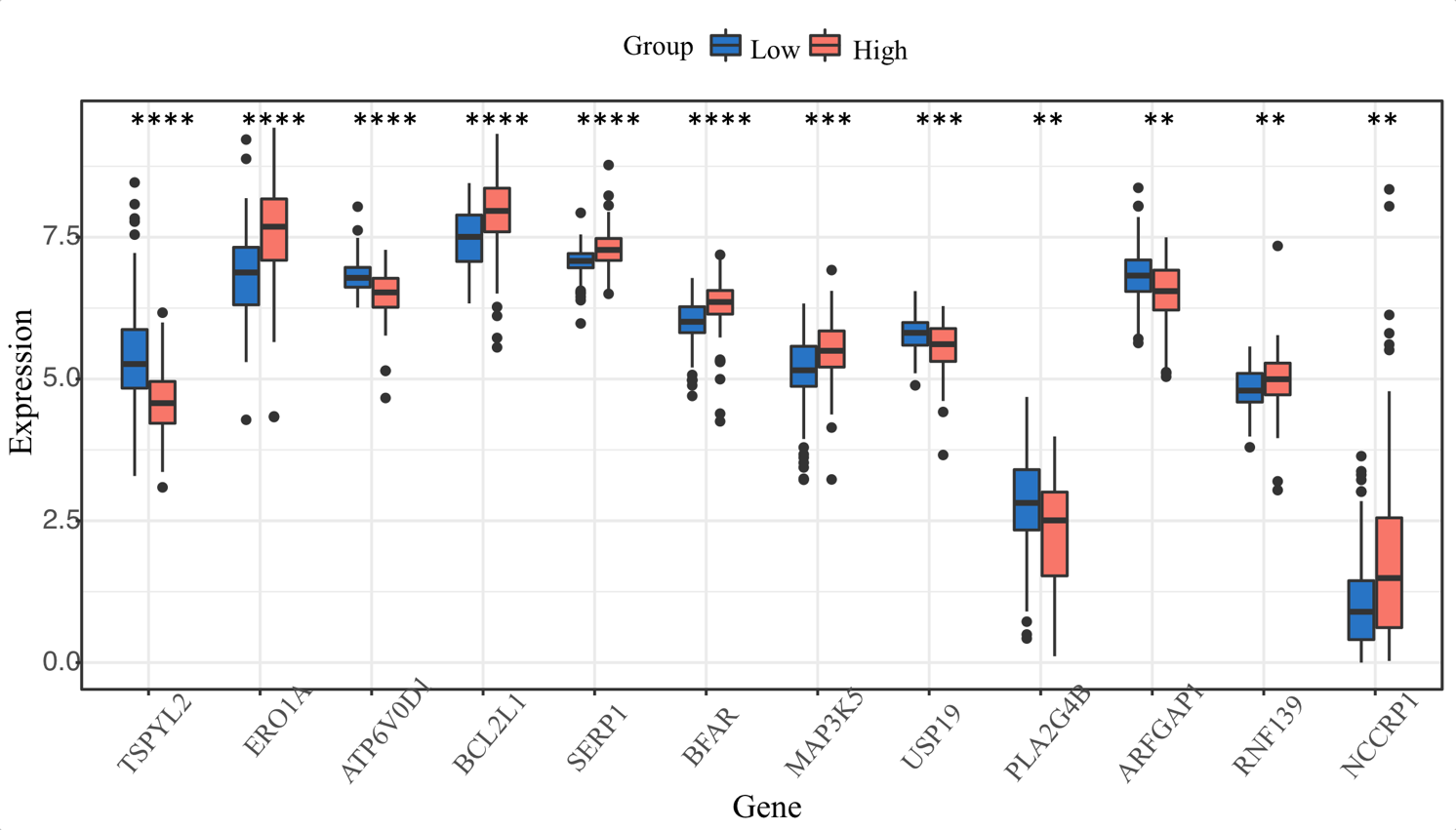


**Supplementary Figure 2.** The differential expression landscape of the 12 prognostic genes between the high- and -low risk clusters.

# Supplementary Tables

**Supplementary Table 1.** 295 unqiue ER stress-related genes obtained from MSigDB v7.4

**Supplementary Table 2.** 99 risk genes to survival of PC patients identified by univariate Cox proportional hazards regression analysis

**Supplementary Table 3.** 56 prognosis-related genes identified by log rank test

**Supplementary Table 4.** GO enrichment analysis of differentially expressed genes (DEGs) between high- and low-risk samples

# Full original source data

# We have uploaded the full original source data and codes used in this study at Jianguoyun and generated a freely accessible link (https://www.jianguoyun.com/p/DYpnVlIQ4qX6CxjQ3ZoFIAA)
